# Exposure-response relationship between ambient PM_2.5_ and SO_2_ with COPD and SAD prevalence among individuals at high risk for COPD in Yunnan Province, China: a threshold analysis

**DOI:** 10.3389/fpubh.2026.1874156

**Published:** 2026-09-01

**Authors:** Jinliang Meng, Geyi Wen, Guoyu Ma, Ruiqi Wang, Yanyan Xu, Yunhui Zhang, Le Cai

**Affiliations:** 1 Yunnan Provincial Key Laboratory of Public Health and Biosafety & School of Public Health, Kunming Medical University, Kunming, Yunnan, China; 2 Department of Pulmonary and Critical Care Medicine, The First People's Hospital of Yunnan Province, The Affiliated Hospital of Kunming University of Science and Technology, Kunming, Yunnan, China; 3 Medical School, Kunming University of Science and Technology, Kunming, Yunnan, China; 4 Faculty of Life Science and Technology, Kunming University of Science and Technology, Kunming, Yunnan, China; 5 Department of Cancer Prevention and Control, Yunnan Cancer Hospital, Kunming, Yunnan, China

**Keywords:** chronic obstructive pulmonary disease, effect threshold, exposure-response relationship, PM_2.5_, sulfur dioxide

## Abstract

**Background:**

Ambient air pollution is a major risk factor for chronic obstructive pulmonary disease (COPD) and small airway dysfunction (SAD). However, evidence on their nonlinear exposure-response relationships and effect thresholds remain insufficient, and most identified thresholds lack clinical relevance evaluation. This study aimed to examine the nonlinear links of PM_2.5_ and SO_2_ exposure with the prevalence of COPD and SAD, identify effect thresholds, and compare clinical characteristics across subgroups stratified by these threshold values.

**Methods:**

A cross-sectional study was conducted in Yunnan Province from 2021 to 2022, enrolling 11,095 permanent residents aged ≥20 years via multistage random cluster sampling. High-risk individuals were screened using COPD-SQ/COPD-PS questionnaires and received standardized pulmonary function testing. COPD was diagnosed per GOLD guidelines. SAD was defined as at least two of maximal mid-expiratory flow (MMEF), forced expiratory flow at 50% of vital capacity (FEF_50_%), and forced expiratory flow at 75% of vital capacity (FEF_75_%) below 65% of predicted values. Restricted cubic spline models were used to assess nonlinear relationships and identify thresholds. Stratification analyses were performed to compare clinical symptoms and lung function between exposure groups, and random forest models were used to evaluate the importance of exposure-related factors.

**Results:**

Among high-risk participants, COPD and SAD prevalence were 27.2 % and 53.6%, respectively. Significant nonlinear exposure-response relationships were observed for both PM_2.5_ and SO_2_ with both outcomes (all *P* < 0.001). The effect thresholds were identified as 21.33 μg/m^3^ for PM_2.5_ and 8.67 μg/m^3^ for SO_2_. Among those with PM_2.5_ > 21.33 μg/m^3^, the prevalence of COPD and SAD was 33.4% and 58.4%, respectively; among those with SO_2_ > 8.67 μg/m^3^, the corresponding prevalence was 30.2% and 57.4% (all *P* < 0.05). In addition, high exposure groups had significantly higher prevalence of cough and dyspnea, as well as → poorer lung function (FEV_1_, FVC, and FEV_1_ /FVC), compared with low exposure groups. Air pollution was the associated factor for both outcomes, exceeding smoking and biomass fuel exposure.

**Conclusion:**

Nonlinear relationships exist between PM_2.5_/SO_2_ exposure and the prevalence of COPD and SAD, with core thresholds of 21.33 μg/m^3^ (PM_2.5_) and 8.67 μg/m^3^ (SO_2_). In high-risk individuals and COPD patients, exposure above these thresholds correlates with more severe respiratory symptoms and poorer lung function, illustrating the thresholds' clinical relevance.

## 1 Background

Chronic obstructive pulmonary disease (COPD), characterized by persistent airflow limitation, is currently among the top three leading causes of death worldwide. Notably, 90% of COPD-related deaths occur in low- and middle-income countries (LMICs) (1, 2), imposing substantial disease and socioeconomic burdens (3). Small airway dysfunction (SAD) is recognized as a critical early stage in the initiation and progression of COPD (4). It often emerges before the onset of overt clinical symptoms or measurable airflow limitation, representing a key window for early prevention and control of chronic respiratory diseases (5).

Ambient air pollution poses a major threat to human health (6–8). Globally, it contributes to an estimated 9 million premature deaths annually, accounting for one in every six deaths (9). It is responsible for 8% of all disability-adjusted life years (DALYs), a proportion exceeding those attributable to hypertension and smoking (10). Mounting evidence implicates air pollution as a leading risk factor for both COPD and SAD (7, 11, 12). The 2023 GOLD report noted that air pollution may account for up to 50% of the overall attributable risk of COPD (12). The European Study of Cohorts for Air Pollution Effects (ESCAPE) demonstrated that higher levels of air pollution correlated with greater impairment of lung function in adults (13). The Acute Exacerbation of Chronic Obstructive Pulmonary Disease in China Registry (ACURE) study identified elevated air pollution as an important risk factor for acute exacerbations of COPD (14). Therefore, in-depth investigation into the effects of air pollutants on COPD and SAD carries critical public health significance.

Despite these advances, critical knowledge gaps and controversies persist in the existing evidence. First, while robust evidence links air pollutants to COPD incidence, acute exacerbations, and mortality (12–14), research on the relationship between air pollutants and SAD remains limited. Evidence on nonlinear exposure-response relationships between pollutant exposure and health outcomes is scarce. This is particularly true for the identification of specific effect thresholds within real-world exposure ranges (15). Second, most existing studies rely on ecological environmental data and lack in-depth analyses of population-specific effects, particularly for subgroups defined by sex, age, or ethnicity that may exhibit heightened susceptibility to air pollution. Third, when thresholds have been identified, they are often limited to statistical determination without clinical characterization using patient symptoms and lung function impairment (16, 17). These limitations hinder precise health risk assessment and targeted intervention from a public health perspective.

To address these gaps, the present study builds on our previous epidemiological investigation of COPD in Yunnan Province (18). By integrating monitoring data for fine particulate matter (PM_2.5_) and sulfur dioxide (SO_2_) across Yunnan, we systematically clarified their relationships with COPD and SAD, and assessed the clinical relevance of statistical thresholds against clinical phenotypes. Our primary hypothesis was that exposure to PM_2.5_ and SO_2_ would show nonlinear positive associations with the prevalence of COPD and SAD, with identifiable effect thresholds that are clinically meaningful and consistent across the two outcomes. Our findings provide a scientific basis for innovating environmental health risk assessment methodologies, optimizing early prevention and control strategies for COPD, and implementing precision environmental health management.

## 2 Methods

### 2.1 Study population and study design

This cross-sectional study enrolled permanent residents aged ≥20 years in Yunnan Province, China, using a multistage stratified random cluster sampling approach. A large-scale, representative epidemiological survey of COPD was conducted between 2021 and 2022, with detailed procedures reported previously (18). Briefly, 16 counties were randomly selected from 16 prefecture-level cities in Yunnan, and four townships or villages were further randomly sampled from each county. Participants were stratified by sex and age, and one eligible resident was randomly selected from each household. A total of 11,095 participants aged ≥20 years were finally included.

The inclusion of participants aged ≥20 years in this study was based on three considerations. First, recent COPD epidemiological studies have extended their inclusion to individuals aged 20–40 years to capture airflow obstruction across the full adult age spectrum, rather than limiting to the traditional ≥40 years population (19–22). Second, the GOLD diagnostic criteria do not specify a lower age limit. The commonly used “≥40 years” threshold in epidemiological surveys is a design choice based on prevalence and feasibility, not a diagnostic mandate. Our study therefore followed the disease definition set by the guidelines. Third, although COPD is more common in the older adults, younger patients do develop the disease due to genetic factors, heavy smoking, environmental/occupational exposures, or other susceptibilities. The concept of “young COPD” has been formally proposed and is widely recognized, typically defined as individuals aged 20–50 years who meet the diagnostic criteria for COPD (23). Collectively, these considerations support the scientific and clinical rationale for adopting ≥20 years as the inclusion criterion in our study.

This study was approved by the Ethics Committee of the First People's Hospital of Yunnan Province (Approval No. KHLL2022-KY141-C-1). Written informed consent was obtained from all participants prior to enrollment.

A stratified multistage cluster sampling strategy was adopted to recruit study participants. The preliminary sample size was calculated via the standard simple random sampling formula below:


$$\begin{array}{l} n=\frac{{\left(Z\alpha /2\right)}^{2}}{{\left(\delta \right)}^{2}}•\pi \left(1-\pi \right)•deff \end{array}$$

Within this formula, α refers to the significance level set to 0.05, δ indicates the margin of error, and π stands for the anticipated disease prevalence. We extracted an overall COPD prevalence of 8.6% among Chinese adults aged ≥20 years from a nationwide cross-sectional survey conducted by Wang et al. (19). With α = 0.05 and δ = 0.01, the unadjusted sample size was calculated as 3,020 individuals. To accommodate the stratified cluster sampling framework, a design effect of 2.0 was incorporated, raising the preliminary target sample to 6,040. We additionally factored in a 20% rate of incomplete or invalid questionnaires due to non-response, which further inflated the minimum required sample size to 7,550. Our final enrollment totaled 11,095 participants, a number well above the pre-specified minimum threshold, guaranteeing sufficient statistical power for all subsequent analyses.

### 2.2 Survey data

An active case-finding strategy for COPD was implemented among high-risk individuals rather than the entire study population. All participants underwent a two-step screening process using validated COPD-PS (24) and COPD-SQ (25) questionnaires to identify those at high risk (scores ≥5 and ≥16, respectively). High-risk individuals then underwent standardized pulmonary function testing in accordance with GOLD guidelines. To control for potential confounding, we implemented measures at multiple levels. At the design stage, we minimized measurement bias through standardized protocols and investigator training. During fieldwork, all survey sites followed a unified quality control procedure to ensure data consistency. In statistical analysis, we adjusted for potential confounders including age, sex, smoking history and biomass fuel. Participants with a pre-bronchodilator FEV_1_/FVC < 0.7 underwent post-bronchodilator spirometry for confirmatory testing. All high-risk individuals completed a comprehensive questionnaire covering sociodemographic characteristics, risk factor exposure, personal medical history, and comorbidities. COPD was diagnosed in line with GOLD guidelines (23).

SAD was defined as at least two of the three following indices being lower than 65% of the predicted value: maximal mid-expiratory flow (MMEF), forced expiratory flow at 50% of vital capacity (FEF_50_ %), and forced expiratory flow at 75% of vital capacity (FEF_75_ %) (26, 27). Pulmonary function reference equations specific to the Chinese population were used in this study (28, 29).

### 2.3 Environmental exposure data

Annual average concentrations of PM_2.5_ and SO_2_ in major cities of Yunnan Province were obtained from the Yunnan Provincial Statistical Yearbook published by the Yunnan Provincial Bureau of Statistics (30). The Yearbook is publicly accessible via the official website of the Yunnan Provincial Bureau of Statistics (http://stats.yn.gov.cn/tjsj/tjnj/). Consistent with prior studies (7, 16), PM_2.5_ and SO_2_ were chosen as representative indicators to assess ambient air pollution exposure. To characterize long-term individual exposure levels, 3-year average concentrations (2019–2021) immediately before the 2021–2022 epidemiological survey were calculated for each participant's residential area and used as the long-term exposure metric. In this study, exposure assessment was performed at the city level as the basic geographic unit, with annual average concentrations representing urban averages derived from all available monitoring stations within each prefecture-level city, as routinely reported in the Yunnan Provincial Statistical Yearbook, with 3-year average concentrations of PM_2.5_ and SO_2_ assigned to all participants residing within each city. Individual exposure estimates were then spatially linked with participant-level questionnaire data to construct a unified analytical database.

### 2.4 Statistical analysis

Statistical analyses were conducted using SPSS version 27.0 (Statistical Product and Service Solutions, IBM Corp., Armonk, NY, USA) and R version 4.5.2 (R Foundation for Statistical Computing, Vienna, Austria). Categorical variables were presented as counts and percentages [*n* (%)]. The Shapiro-Wilk test was used to assess the normality of continuous variables. Non-normally distributed continuous variables were expressed as median with interquartile range (*P*_25_, *P*_75_). The chi-square test was employed to compare differences in rates between two groups, whereas the Mann–Whitney U-test was used for comparisons of non-normally distributed continuous variables between groups. Given the cross-sectional nature of this study, all associations reported are prevalence-based and should not be interpreted as causal or longitudinal risk estimates.

To explore nonlinear exposure–response relationships between ambient PM_2.5_, SO_2_ and two respiratory outcomes (COPD and SAD), we fitted restricted cubic spline (RCS) models adjusted for predefined confounders. Five knots were placed at the 5th, 27.5th, 50th, 72.5th and 95th percentiles of each pollutant's distribution, matching the default configuration of Harrell's rms package in R. These corresponded to 13.67, 21.33, 22.33, 24.67, and 27.33 μg/m^3^ for PM_2.5_, and 5.67, 7.33, 9.67, 10.00, and 10.67 μg/m^3^ for SO_2_. Due to the continuity constraints imposed on the spline function at the knots, the confidence bands naturally narrow at these locations; when an identified threshold coincides with a knot, this manifests as a narrower confidence interval at that specific point (31). Smoking status was coded as a binary variable (1 = never smoker, 2 = former/current smoker). Missing data for covariates were minimal (< 5% for all variables) and were handled by complete-case analysis, as excluding these cases did not materially affect the sample size or the precision of the estimates. All covariates (age, sex, residential altitude, smoking status, and household biomass fuel exposure) were simultaneously included in the regression models as prespecified confounders. Likelihood ratio tests were adopted to evaluate nonlinearity by comparing linear models against spline models; a nonlinear *P* < 0.05 indicated a statistically significant curved association. Inflection points at which the pollutant–disease association showed a marked change were identified from the fitted RCS curves and defined as exposure effect thresholds. Thresholds were identified using an algorithm-based approach from the fitted restricted cubic spline models. Specifically, we extracted the adjusted *OR* curve from each model and identified the exposure concentration at which the logit (*OR*) crossed 0 (i.e., *OR* = 1), using point-wise predictions from the fitted model to determine the exact x-coordinate of the crossing point.

We stratified participants into high- and low-exposure groups based on these thresholds to compare clinical features across subgroups, including respiratory symptoms and pulmonary function indices. Random forest models were further built to quantify and visualize the relative importance of multiple exposure-related factors, generating variable importance ranking plots. Each random forest contained 500 decision trees, with mtry set to the package default (the square root of the total predictors). Node splitting was performed using the Gini impurity index. To handle potential outcome class imbalance, stratified bootstrap sampling implemented in the randomForest R package was applied to preserve the original case–control ratio within every resample. Variable importance was quantified via the mean decrease in Gini impurity and displayed in ranking figures.

## 3 Results

### 3.1 Univariate analysis of COPD and SAD prevalence among individuals at high risk for COPD in relation to PM_2.5_ or SO_2_ exposure

A total of 11,095 participants were enrolled in this study, of whom 2,252 (20.3%) were identified as high-risk individuals for COPD. Among these 2,252 high-risk individuals, 612 (27.2%) were diagnosed with COPD and 1,206 (53.6%) with SAD. As shown in Table 1, among the high-risk population, the prevalence of COPD was significantly higher in males than in females (*P* < 0.001), while no significant sex difference was observed for SAD prevalence (*P* = 0.505). Significant differences in both COPD and SAD prevalence were observed across age groups and altitude regions (all *P* < 0.05). No significant ethnic differences were found in the prevalence of either COPD (*P* = 0.228) or SAD (*P* = 0.310).

**Table 1:** Comparison of baseline characteristics and PM_2.5_ or SO_2_ exposure status in COPD and SAD populations [*n* (%), *M* (*P*_25_, *P*_75_)].

Variables	Classification	Total	COPD		*χ^2^/Z* statistic	*P*-value	SAD		*χ^2^/Z* statistic	*P*-value
			No	Yes			No	Yes		
Gender	Male	1,463 (65.0)	980 (67.0)	483 (33.0)	71.925	< 0.001^*^	672 (64.2)	791 (65.6)	0.445	0.505
	Female	789 (35.0)	660 (83.7)	129 (16.3)			374 (35.8)	415 (34.4)		
Age (years)	20–49	213 (9.5)	194 (91.1)	19 (8.9)^c^	62.579	< 0.001^*^	128 (12.2)	85 (7.0)^c^	26.994	< 0.001^*^
	50–59	536 (23.8)	407 (75.9)	129 (24.1)^b^			260 (24.9)	276 (22.9)^b^		
	60–69	854 (37.9)	622 (72.8)	232 (27.2)^b^			397 (38)	457 (37.9)^a^		
	≥70	649 (28.8)	417 (64.3)	232 (35.7)^a^			261 (25)	388 (32.2)^a^		
Ethnicity	Han	1,738 (77.2)	1,255 (72.2)	483 (27.8)	1.454	0.228	798 (76.3)	940 (77.9)	0.869	0.310
	Ethnic minorities	514 (22.8)	385 (74.9)	129 (25.1)			248 (23.7)	266 (22.1)		
Elevation	Low	783 (34.8)	576 (73.6)	207 (26.4)^a^	8.495	0.014^*^	379 (36.2)	404 (33.5)^b^	18.688	< 0.001^*^
	Medium	1,313 (58.3)	936 (71.3)	377 (28.7)^a^			572 (54.7)	741 (61.4)^a^		
	High	156 (6.9)	128 (82.1)	28 (17.9)^b^			95 (9.1)	61 (5.1)^c^		
PM _2.5_ (μg/m^3^)		21.00 (21.33,24.67)	21.33 (20.67,24.67)	24.00 (21.33,24.67)	−5.045	< 0.001^*^	21.33 (19.67,24.67)	24.00 (21.33,24.67)	−4.652	< 0.001^*^
SO_2_ (μg/m^3^)		8.67 (6.67,10.00)	9.33 (6.67,10.00)	9.67 (8.33,10.00)	−4.499	< 0.001^*^	9.00 (6.67,10.00)	9.67 (8.33,10.00)	– 5.286	< 0.001^*^

(1) Different superscript letters (a, b, c) indicate statistically significant differences between groups based on Bonferroni pairwise comparisons; (2) An asterisk (^*^) denotes statistical significance at *P* < 0.05.

Regarding pollutant exposure, the COPD group had significantly higher exposure levels of both PM_2.5_ and SO_2_ compared with the non-COPD group (both *P* < 0.001). Similarly, the SAD group exhibited significantly higher exposure levels of PM_2.5_ and SO_2_ than the non-SAD group (both *P* < 0.001).

### 3.2 Exposure-response associations of PM_2.5_ and SO_2_ with COPD and SAD prevalence among individuals at high risk for COPD

**Figure 1:**
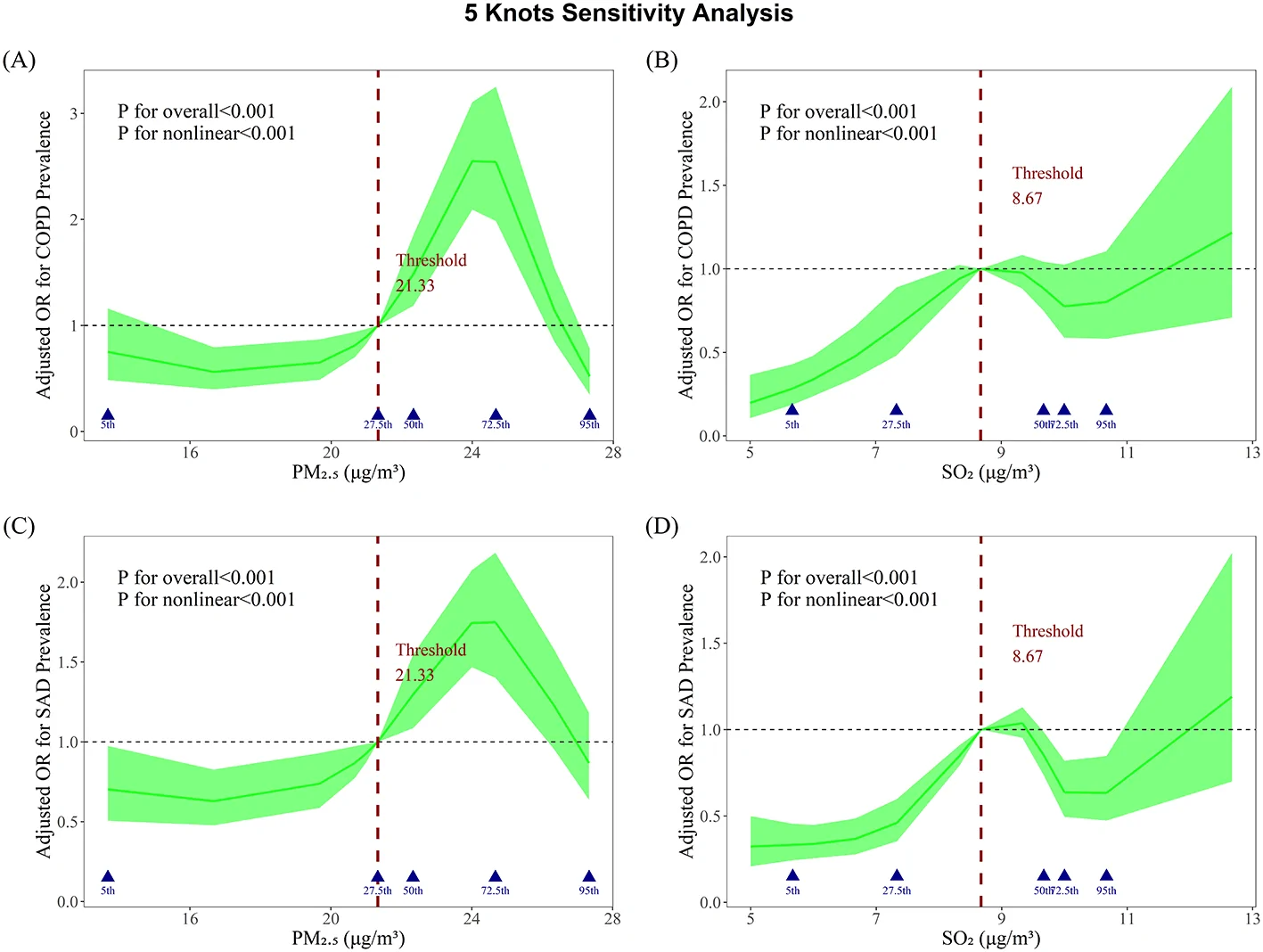
Exposure-response curves for PM_2.5_ and SO_2_with the prevalence of COPD and SAD among the high-risk population for COPD using restricted cubic spline models. The solid lines represent the adjusted odds ratios, with shaded areas indicating the 95% confidence intervals. Models were adjusted for age, sex, residential altitude, smoking status, and household biomass fuel exposure. Vertical dashed lines mark the identified threshold concentrations: **(A)** PM_2.5_ threshold at approximately 21.33 μg/m^3^ for COPD; **(B)** SO_2_ threshold at approximately 8.67 μg/m^3^ for COPD; **(C)** PM_2.5_ threshold at approximately 21.33 μg/m^3^ for SAD; **(D)** SO_2_ threshold at approximately 8.67 μg/m^3^ for SAD. The analysis included 2,252 high-risk individuals.

Among the high-risk population (*n* = 2,252), PM_2.5_ exposure ranged from 13.67 to 27.33 μg/m^3^ (median: 22.33 μg/m^3^; 95th percentile: 27.33 μg/m^3^; 97.5th percentile: 27.33 μg/m^3^), and SO_2_ exposure ranged from 5.00 to 12.67 μg/m^3^ (median: 8.67 μg/m^3^; 95th percentile: 10.67 μg/m^3^; 97.5th percentile: 12.67 μg/m^3^). The exposure distributions are presented in Supplementary Figure S4. The prevalence of COPD and SAD across exposure deciles is presented in Supplementary Tables S3, S4. For both PM_2.5_ and SO_2_, the prevalence increased progressively from decile 1 through decile 8, but irregular fluctuations appeared in the highest deciles (deciles 9–10). After adjustment for age, sex, residential altitude, smoking status, and household biomass fuel exposure, analyses for relationships between PM_2.5_, SO_2_, and COPD demonstrated that prevalence of COPD among individuals at high risk for COPD increased significantly when PM_2.5_ exceeded 21.33 μg/m^3^ (nonlinear *P* < 0.001; Figure 1A) and when SO_2_ exceeded 8.67 μg/m^3^ (nonlinear *P* < 0.001; Figure 1B).

Similarly, after adjustment for age, sex, residential altitude, smoking status, and household biomass fuel exposure, the prevalence of SAD among individuals at high risk for COPD increased significantly when PM_2.5_ exceeded 21.33 μg/m^3^ (nonlinear *P* < 0.001; Figure 1C) and when SO_2_ exceeded 8.67 μg/m^3^ (nonlinear *P* < 0.001; Figure 1D). To assess the robustness of the threshold estimates to knot specification, we conducted sensitivity analyses using alternative knot configurations (three knots at 10th, 50th, and 90th percentiles; four knots at 5th, 35th, 65th, and 95th percentiles; and six knots at 5th, 23rd, 41st, 59th, 77th, and 95th percentiles). Across all specifications, the threshold estimates remained identical: 21.33 μg/m^3^ for PM_2.5_ and 8.67 μg/m^3^ for SO_2_ for both outcomes (Supplementary Table S1; Supplementary Figures S1–S3).

To evaluate whether the observed upward rebound at the highest exposure levels was robust or attributable to sparse observations in the upper tail, we performed spline analyses truncated at the 90th, 95th, and 97.5th percentiles of the exposure distribution. Due to the discrete nature of the exposure data, the 95th and 97.5th percentiles coincided with the maximum observed values for PM_2.5_ (27.33 μg/m^3^), yielding no meaningful reduction in sample size; for SO_2_, the 95th percentile retained 2,176 participants, identical to the 90th. Therefore, we focused on the 90th percentile truncation (Supplementary Figure S5). The 90th percentile truncation revealed that the upward rebound was eliminated, with the COPD curve showing a stepwise increasing pattern and the SAD curve showing a fluctuating upward trend that leveled off without any sharp rebound at extreme concentrations. These findings suggest that the tail rebound is likely attributable to sparse observations in the upper tail rather than a genuine biological phenomenon.

To assess the stability of the threshold estimates against sampling variability, we performed a bootstrap internal validation with 500 resamples. The bootstrap means were highly consistent: 22.26 μg/m^3^ (SD = 0.51) for PM_2.5_ → COPD, 9.63 μg/m^3^ (SD = 0.13) for SO_2_ → COPD, 22.29 μg/m^3^ (SD = 0.58) for PM_2.5_ → SAD, and 9.63 μg/m^3^ (SD = 0.19) for SO_2_ → SAD, confirming that the thresholds are stable and not substantially influenced by sampling variability (Supplementary Table S5; Supplementary Figure S6).

For subsequent stratified analyses, participants were stratified using the effect thresholds for COPD (PM_2.5_: 21.33 μg/m^3^; SO_2_: 8.67 μg/m^3^) based on the following considerations: first, COPD was the primary outcome of this study, and using its thresholds for stratification was most consistent with the study design; second, thresholds for COPD and SAD were highly consistent (difference < 0.2 μg/m^3^), such that using either set of thresholds had minimal impact on actual grouping results. Accordingly, pollutant levels were categorized as low or high exposure based on these thresholds.

### 3.3 Clinical characterization by PM_2.5_ or SO_2_ exposure levels among individuals at high risk for COPD

As shown in Table 2, for sociodemographic characteristics, the proportion of males was significantly higher in the high PM_2.5_ exposure group (*P* = 0.010), whereas no significant sex difference was observed between SO_2_ exposure groups (*P* = 0.734). Age distribution did not differ significantly between PM_2.5_ exposure groups (*P*= 0.205), but the high SO_2_ exposure group had a higher proportion of participants aged ≥70 years (32.2%, *P* < 0.001). BMI classification showed no significant difference between PM_2.5_ exposure groups (*P* = 0.386) but differed significantly between SO_2_ exposure groups (*P* = 0.017). Ethnic distribution differed significantly across both exposure groups (both *P* < 0.001): the high PM_2.5_ exposure group had a lower proportion of Han individuals, whereas the high SO_2_ exposure group had a higher proportion. No significant differences were observed in smoking status. For biomass fuel use, both the high PM_2.5_ and high SO_2_ exposure groups had lower proportions of biomass fuel users (*P* = 0.004 and *P* = 0.002, respectively).

**Table 2:** Baseline characteristics by PM_2.5_ or SO_2_ exposure levels among individuals at high risk for COPD [*n* (%), *M* (*P*_25_, *P*_75_)].

Variables	Classification	Total	PM_2.5_		*χ^2^/Z* statistic	*P*-value	SO_2_		*χ^2^/Z* statistic	*P*-value
			Low Exposure ( ≤ 21.33 μg/m^3^)	High Exposure (> 21.33 μg/m^3^)			Low Exposure ( ≤ 8.67 μg/m^3^)	High Exposure		
Gender	Male	1,463 (65.0)	684 (62.3)	779 (67.5)	6.708^a^	0.010^*^	664 (64.6)	799 (65.3)	0.116^a^	0.734
	Female	789 (35.0)	414 (37.7)	375 (32.5)			364 (35.4)	425 (34.7)		
Age group	20–49	213 (9.5)	94 (8.6)	119 (10.3)	4.586^a^	0.205	119 (11.6)	94 (7.7)	2.386^a^	< 0.001^*^
	50–59	536 (23.8)	248 (22.6)	288 (25.0)			260 (25.3)	276 (22.5)		
	60–69	854 (37.9)	430 (39.2)	424 (36.7)			394 (38.3)	460 (37.6)		
	≥70	649 (28.8)	326 (29.7)	323 (28.0)			255 (24.8)	394 (32.2)		
BMI (kg/m^2^)	< 18.5	177 (7.9)	82 (7.5)	95 (8.2)	3.042^a^	0.386	86 (8.4)	91 (7.4)	10.204^a^	0.017^*^
	18.5–24.9	1,421 (63.1)	708 (64.5)	713 (61.8)			620 (60.3)	801 (65.4)		
	25.5–29.9	545 (24.2)	262 (23.9)	283 (24.5)			259 (25.2)	286 (23.4)		
	≥30	109 (4.8)	46 (4.2)	63 (5.5)			63 (6.1)	46 (3.8)		
Ethnicity	Han	1,738 (77.2)	918 (83.6)	820 (71.1)	50.305^a^	< 0.001^*^	682 (66.3)	1,056 (86.3)	126.019a	< 0.001^*^
	Ethnic minorities	514 (22.8)	180 (16.4)	334 (28.9)			346 (33.7)	168 (13.7)		
Smoking status	Never smoking	953 (42.3)	467 (42.5)	486 (42.1)	0.040^a^	0.841	441 (42.9)	512 (41.8)	0.261^a^	0.609
	Previous smoking	1,299 (57.7)	631 (57.5)	668 (57.9)			587 (57.1)	712 (58.2)		
Biomass fuel exposure	No	1,306 (58.0)	603 (54.9)	703 (60.9)	8.316^a^	0.004^*^	560 (54.5)	746 (60.9)	9.610^a^	0.002^*^
	Yes	946 (42.0)	495 (45.1)	451 (39.1)			468 (45.5)	478 (39.1)		
Cough	No	937 (41.6)	485 (44.2)	452 (39.2)	5.797^a^	0.016^*^	424 (41.2)	513 (41.9)	0.102^a^	0.749
	Yes	1,315 (58.4)	613 (55.8)	702 (60.8)			604 (58.8)	711 (58.1)		
Dyspnea	No	448 (19.9)	227 (20.7)	221 (19.2)	0.819^a^	0.365	225 (21.9)	223 (18.2)	4.718^a^	0.030^*^
	Yes	1,804 (80.1)	871 (79.3)	933 (80.8)			803 (78.1)	1,001 (81.8)		
Presence of activity limitation attributable to respiratory symptoms	No	1,210 (53.7)	577 (52.6)	633 (54.9)	1.200^a^	0.273	559 (54.4)	651 (53.2)	0.319^a^	0.572
	Yes	1,042 (46.3)	521 (47.4)	521 (45.1)			469 (45.6)	573 (46.8)		
COPD	No	1,640 (72.8)	871 (79.3)	769 (66.6)	45.771^a^	< 0.001^*^	786 (76.5)	854 (69.8)	12.628^a^	< 0.001^*^
	Yes	612 (27.2)	227 (20.7)	385 (33.4)			242 (23.5)	370 (30.2)		
SAD	No	1,046 (46.4)	566 (51.5)	480 (41.6)	22.412^a^	< 0.001^*^	524 (51.0)	522 (42.6)	15.571^a^	< 0.001^*^
	Yes	1,206 (53.6)	532 (48.5)	674 (58.4)			504 (49.0)	702 (57.4)		
FEV_1_ (L)		2.3 (1.73,2.84)	2.38 (1.87,2.92)	2.19 (1.61,2.78)	−5.805^b^	< 0.001^*^	2.37 (1.82,2.91)	2.22 (1.64,2.8)	−4.474^b^	< 0.001^*^
FEV_1_%pred (%)		95.65 (78.38,110.41)	100 (84.7,115.23)	91.94 (71.14,105.53)	−8.87^b^	< 0.001^*^	97.89 (82.52,111.64)	94.16 (74.73,109.23)	−3.66^b^	< 0.001^*^
FEV_1_/FVC (%)		3.11 (2.49,3.8)	3.19 (2.56,3.85)	3.04 (2.41,3.74)	−3.76^b^	< 0.001^*^	3.19 (2.59,3.86)	3.05 (2.42,3.72)	−4.298^b^	< 0.001^*^
FVC (L)		101.93 (88.41,116.02)	105.42 (92.26,120.26)	99.2 (84.51,112.06)	−7.864^b^	< 0.001^*^	103.72 (90.27,117.13)	100.45 (86.35,115.2)	−3.728^b^	< 0.001^*^
FVC%pred (%)		75.06 (66.81,80.28)	75.81 (69.4,80.84)	74.06 (63.05,79.78)	−5.698^b^	< 0.001^*^	75.53 (68.82,80.22)	74.37 (65.01,80.4)	−2.026^b^	0.043^*^

(1) Superscript a indicates Chi-square test; superscript b indicates rank-sum test; (2) An asterisk (^*^) denotes statistical significance at *P* < 0.05; (3) abbreviation: BMI, body mass index; COPD, Chronic Obstructive Pulmonary Disease; SAD, Small Airway Dysfunction; FEV_1_ and FEV_1_%pred, forced expiratory volume in the first second and its percentage of the predicted value; FVC and FVC%pred, forced vital capacity and its percentage of the predicted value.

In terms of symptoms, cough was more prevalent in the high PM_2.5_ exposure group (*P* = 0.016) but did not differ significantly between SO_2_ exposure groups (*P* = 0.749). Dyspnea showed no significant difference between PM_2.5_ exposure groups (*P* = 0.365) but was more common in the high SO_2_ exposure group (*P* = 0.030). No significant differences were observed in activity limitation attributable to respiratory symptoms.

Regarding disease prevalence, both the high PM_2.5_ and high SO_2_ exposure groups had significantly higher prevalence of COPD and SAD (all *P* < 0.001). For lung function, the high PM_2.5_ and high SO_2_ exposure groups exhibited significantly lower values of FEV_1_, FEV_1_%pred, FEV_1_/FVC, FVC, and FVC%pred compared with the low exposure groups (all *P* < 0.001).

In a sensitivity analysis restricted to participants aged ≥40 years (adjusted for sex, residential altitude, smoking status, and household biomass fuel exposure), the threshold estimates were 22.33 μg/m^3^ for PM_2.5_ and 9.67 μg/m^3^ for SO_2_ (Supplementary Table S6). In the ≥40 years subgroup, the prevalence of COPD and SAD was 27.7% and 54.1%, respectively. Stratified by the PM_2.5_ threshold (21.33 μg/m^3^), COPD prevalence in the high exposure group was 34.2%, significantly higher than that in the low exposure group (21.1%, *P* < 0.001); SAD prevalence was 59.6% in the high exposure group versus 48.5% in the low exposure group (*P* < 0.001). Stratified by the SO_2_ threshold (8.67 μg/m^3^), COPD prevalence in the high exposure group was 30.4%, significantly higher than that in the low exposure group (24.5%, *P* = 0.002); SAD prevalence was 57.4% in the high exposure group versus 50.1% in the low-exposure group (*P* < 0.001).

In a sensitivity analysis excluding smoking from the covariate adjustment (adjusted for age, sex, residential altitude, and household biomass fuel exposure), the threshold estimates remained unchanged (PM_2.5_ 21.33 μg/m^3^; SO_2_ 8.67 μg/m^3^; Supplementary Table S6), confirming that our findings are robust to different covariate specifications.

### 3.4 Clinical characterization by PM_2.5_ or SO_2_ exposure levels in patients with COPD

Table 3 shows differences in characteristics among COPD patients stratified by PM_2.5_ and SO_2_ exposure levels. No significant differences were observed in sex, age, BMI classification, or smoking status between high and low exposure groups for either PM_2.5_ or SO_2_. Ethnic distribution did not differ significantly between PM_2.5_ exposure groups, but the high SO_2_ exposure group had a significantly higher proportion of Han individuals (*P* < 0.001). For biomass fuel use, no significant difference was found between PM_2.5_ exposure groups, whereas the high SO_2_ exposure group had a significantly lower proportion of biomass fuel users (*P* = 0.001).

**Table 3:** Baseline characteristics by PM_2.5_ or SO_2_ exposure levels in patients with COPD [*n* (%), *M* (*P*_25_, *P*_75_)].

Variables	Classification	Total	PM_2.5_		*χ^2^/Z* statistic	*P*-value	SO_2_		*χ^2^/Z* statistic	*P*-value
			Low Exposure ( ≤ 21.33 μg/m^3^)	High Exposure(> 21.33 μg/m^3^)			Low Exposure ( ≤ 8.67 μg/m^3^)	High Exposure (> 8.67 μg/m^3^)		
Gender	Male	483 (78.9)	175 (77.1)	308 (80.0)	0.726^a^	0.394	197 (81.7)	286 (77.1)	1.902^a^	0.168
	Female	129 (21.1)	52 (22.9)	77 (20.0)			44 (18.3)	85 (22.9)		
Age group	20–49	19 (3.1)	11 (4.8)	8 (2.1)	6.429^a^	0.092	7 (2.9)	12 (3.2)	1.354^a^	0.716
	50–59	129 (21.1)	39 (17.2)	90 (23.4)			54 (22.4)	75 (20.2)		
	60–69	232 (37.9)	90 (39.6)	142 (36.9)			95 (39.4)	137 (36.9)		
	≥70	232 (37.9)	87 (38.3)	145 (37.7)			85 (35.3)	147 (39.6)		
BMI (kg/m^2^)	< 18.5	73 (11.9)	19 (8.4)	54 (14)	4.366^a^	0.225	36 (14.9)	37 (10)	6.837^a^	0.077
	18.5–24.9	410 (67)	158 (69.6)	252 (65.5)			147 (61)	263 (70.9)		
	25.5–29.9	109 (17.8)	42 (18.5)	67 (17.4)			49 (20.3)	60 (16.2)		
	≥30	20 (3.3)	8 (3.5)	12 (3.1)			9 (3.7)	11 (3.0)		
Ethnicity	Han	483 (78.9)	187 (82.4)	296 (76.9)	2.593^a^	0.107	169 (70.1)	314 (84.6)	18.494^a^	< 0.001^*^
	Ethnic minorities	129 (21.1)	40 (17.6)	89 (23.1)			72 (29.9)	57 (15.4)		
Smoking status	Never smoking	189 (30.9)	68 (30)	121 (31.4)	0.145^a^	0.703	69 (28.6)	120 (32.3)	0.944^a^	0.331
	Previous smoking	423 (69.1)	159 (70)	264 (68.6)			172 (71.4)	251 (67.7)		
Biomass fuel exposure	No	385 (62.9)	135 (59.5)	250 (64.9)	1.827^a^	0.176	133 (55.2)	252 (67.9)	10.159^a^	0.001^*^
	Yes	227 (37.1)	92 (40.5)	135 (35.1)			108 (44.8)	119 (32.1)		
Cough	No	245 (40.0)	103 (45.4)	142 (36.9)	4.289^a^	0.038^*^	100 (41.5)	145 (39.1)	0.354^a^	0.552
	Yes	367 (60.0)	124 (54.6)	243 (63.1)			141 (58.5)	226 (60.9)		
Dyspnea	No	77 (12.6)	26 (11.5)	51 (13.2)	0.417^a^	0.580	40 (16.6)	37 (10.0)	5.829^a^	0.016^*^
	Yes	535 (87.4)	201 (88.5)	334 (86.8)			201 (83.4)	334 (90.0)		
Presence of activity limitation attributable to respiratory symptoms	No	379 (61.9)	141 (62.1)	238 (61.8)	0.005^a^	0.942	142 (58.9)	237 (63.9)	1.525^a^	0.217
	Yes	233 (38.1)	86 (37.9)	147 (38.2)			99 (41.1)	134 (36.1)		
GOLD stages	GOLD I	184 (30.1)	87 (38.3)	97 (25.2)	12.586^a^	0.002^*^	84 (34.9)	100 (27.0)	7.974^a^	0.019^*^
	GOLD II	255 (41.7)	88 (38.8)	167 (43.4)			103 (42.7)	152 (41.0)		
	GOLD III/VI	173 (28.3)	52 (22.9)	121 (31.4)			54 (22.4)	119 (32.1)		
FEV_1_ (L)		1.52 (1.08,2.07)	1.67 (1.23,2.23)	1.47 (1.03,1.92)	−3.738^b^	< 0.001^*^	1.67 (1.28,2.2)	1.44 (1.03,1.93)	−3.819^b^	< 0.001^*^
FEV_1_%pred (%)		65.56 (46.82,83.61)	71.68 (53.53,87.76)	61.11 (45.77,80.59)	−3.949^b^	< 0.001^*^	69.14 (52.38,86.57)	63.5 (45.01,81.63)	−2.422^b^	0.015^*^
FEV_1_/FVC (%)		2.74 (2.13,3.44)	2.97 (2.33,3.6)	2.64 (2.07,3.32)	−3.774^b^	< 0.001^*^	2.96 (2.36,3.6)	2.64 (2.05,3.32)	−3.762^b^	< 0.001^*^
FVC (L)		89.95 (73.12,106.48)	96.04 (80.53,110.19)	87.84 (70.64,104.15)	−3.755^b^	< 0.001^*^	93.47 (76.6,107.52)	87.89 (71.77,105.05)	−2.242^b^	0.025^*^
FVC%pred (%)		57.55 (48.04,64.29)	59.06 (49.84,65.13)	56.15 (47.17,63.18)	−2.611^b^	0.009^*^	58.92 (48.8,64.89)	56.35 (47.61,63.8)	−2.056^b^	0.040^*^

(1) Superscript a indicates Chi-square test; superscript b indicates rank-sum test; (2) An asterisk (^*^) denotes statistical significance at *P* < 0.05; (3) abbreviation: BMI, body mass index; COPD, Chronic Obstructive Pulmonary Disease; SAD, Small Airway Dysfunction; FEV_1_ and FEV_1_%pred, forced expiratory volume in the first second and its percentage of the predicted value; FVC and FVC%pred, forced vital capacity and its percentage of the predicted value.

In terms of symptoms, the proportion of patients with cough was significantly higher in the high PM_2.5_ exposure group (*P* = 0.038), but no significant difference was observed between SO_2_ exposure groups (*P* = 0.552). Dyspnea showed no significant difference between PM_2.5_ exposure groups (*P* = 0.580) but was more common in the high SO exposure group (*P* = 0.016). No significant differences were found in activity limitation attributable to respiratory symptoms between exposure groups for either pollutant.

Regarding disease severity, the high PM_2.5_ and high SO_2_ exposure groups had significantly lower values of FEV_1_, FEV_1_%pred, FEV_1_/FVC, FVC, and FVC%pred compared with the low exposure groups (all *P* < 0.05). Furthermore, both high exposure groups had more advanced GOLD stages (PM_2.5_: *P* = 0.002; SO_2_: *P* = 0.019).

### 3.5 Random forest assessment of exposure factor importance

**Figure 2:**
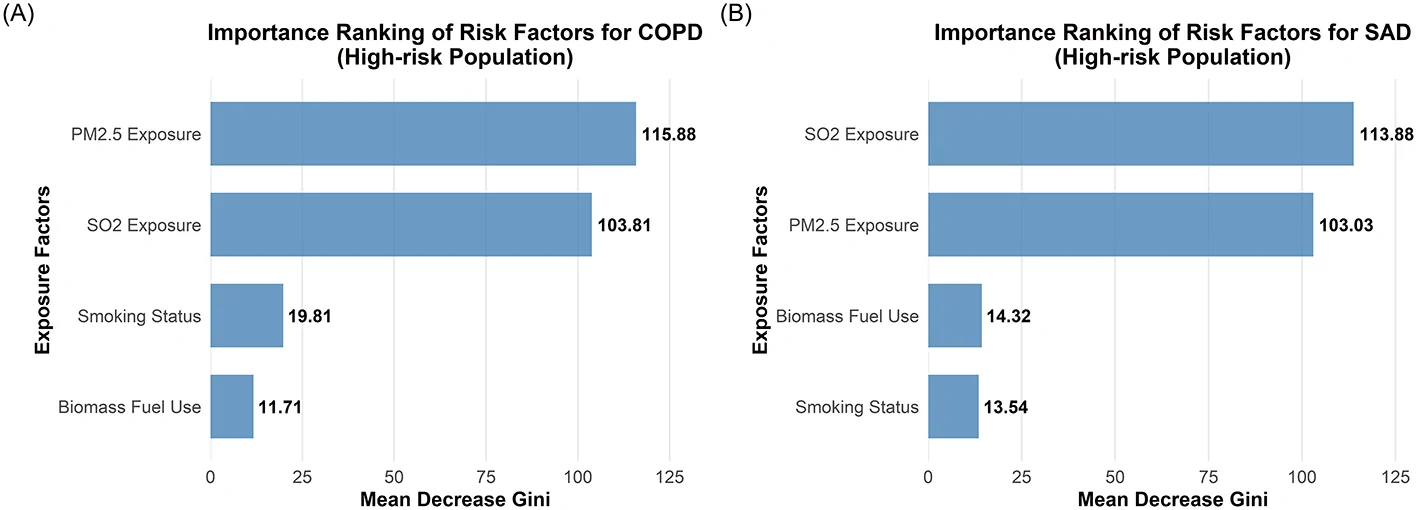
Relative importance of associated exposure factors for COPD and SAD based on random forest analysis. The figure shows the ranked importance of PM_2.5_, SO_2_, smoking, and biomass fuel exposure as predictors of COPD and SAD. Variable importance was measured by the mean decrease in Gini impurity. Higher values indicate greater contribution to the model's predictive accuracy. The analysis was performed on the high-risk population (*n* = 2,252).

Using COPD and SAD as dependent variables, random forest models were applied to calculate the mean decrease in Gini impurity for four exposure factors: PM_2.5_, SO_2_, smoking status, and biomass fuel use. These factors were then ranked to assess their relative importance for the two diseases (Figure 2). In both COPD and SAD models, the order of contribution was consistent: PM_2.5_ > SO_2_ > smoking status > biomass fuel use (Figure 2).

## 4 Discussion

### 4.1 Principal findings and significance

Previous research has established ambient air pollution as a critical exposure factor in the development and progression of both COPD and SAD (9–12). Identifying specific exposure thresholds are therefore crucial for devising precise prevention strategies. However, systematic investigations into such thresholds for COPD and SAD remain scarce. To address this gap, we enrolled a high-risk COPD population in Yunnan Province and systematically evaluated the nonlinear exposure–response relationships between PM_2.5_ and SO_2_ and these two diseases. Our principal findings are as follows. First, we confirmed that exposure to PM_2.5_ and SO_2_ is a significant associated factor for COPD and SAD. Second, we demonstrated nonlinear exposure–response relationships between pollutant concentrations and disease prevalence. Third, we identified effect thresholds for PM_2.5_ (21.33 μg/m^3^) and SO_2_ (8.67 μg/m^3^). Fourth, threshold-based stratification showed that high exposure groups had more severe clinical symptoms, higher disease prevalence, and poorer lung function, supporting the clinical relevance of these thresholds. Finally, random forest analysis indicated that air pollution was the most important contributor to both COPD and SAD, exceeding traditional exposure factors.

To the best of our knowledge, this study is the first to establish clinically relevant effect thresholds for PM_2.5_ and SO_2_ exposure in a COPD high-risk population, thereby filling a critical evidence gap. These thresholds offer quantitative benchmarks for individualized respiratory health risk assessments. Furthermore, they provide actionable scientific evidence for policymakers to set differentiated air quality targets and for healthcare providers to implement stratified prevention strategies. Air pollution ranked prominently in our random forest models. This underscores the imperative of integrating environmental monitoring data into COPD and SAD control programs. Such integration would advance a framework for precision environmental health management.

### 4.2 Pathogenic mechanisms and susceptible populations of PM_2.5_ and SO_2_

The role of PM_2.5_ and SO_2_ as pathogenic risk factors for COPD and SAD is well-documented (19, 32–35) and aligns with our findings. Mechanistic studies have elucidated pathways through which these pollutants contribute to COPD pathogenesis. Recent evidence indicates that PM_2.5_ promotes lung parenchymal destruction, airway remodeling, and small airway dysfunction via oxidative stress, inflammatory cascades, mitochondrial impairment, ferroptosis, and epigenetic modifications, ultimately leading to emphysema and airway obstruction (36–39). SO_2_ and its hydrated form, sulfite, create an acidic milieu in the airways, inducing injury predominantly through oxidative and inflammatory pathways. In animal models, such injury fosters airway remodeling, mucus hypersecretion, and lung function decline (40–42). Moreover, SO_2_ and PM_2.5_ exhibit synergistic toxicity, mutually amplifying inflammatory and oxidative stress, thereby elevating the risk of both COPD onset and acute exacerbations (39, 41, 43, 44).

It is important to note that the health effects of air pollution are modulated by individual characteristics such as age, sex, and pre-existing conditions. For instance, a Korean study of hospitalized COPD patients found that the hospitalization risk associated with PM_10_ exposure was higher in males (*OR* = 1.012, 95% CI: 1.005–1.019) and more pronounced in those aged ≥ 65 years (45). Underlying conditions also heighten susceptibility. The ACURE study reported that patients with a prior-year history of acute exacerbations or a CAT score ≥10 were more vulnerable to PM_2.5_ exposure (14). Collectively, these findings underscore the need for stricter protective measures for susceptible subpopulations.

### 4.3 Associations of PM_2.5_ and SO_2_ exposure with COPD and SAD prevalence

Our study confirmed a monotonic increase in COPD and SAD prevalence with rising PM_2.5_ and SO_2_ concentrations, consistent with recent large-scale investigations. For example, Xing et al. (39) demonstrated nonlinear exposure-response relationships between both total PM_2.5_ mass and its constituents and COPD incidence in the UK Biobank, noting synergistic effects with genetic predisposition Similarly, a 15-year Korean cohort study of 5.5 million individuals by Xing et al. (39) reinforced the nonlinear impact of PM_2.5_ on COPD Regarding SAD, a national Chinese cross-sectional study by Zhu et al. found long-term PM_2.5_ exposure to be significantly associated with increased SAD risk (*OR* = 1.18, 95% CI: 1.08–1.28) (46).

The nonlinear relationship between SO_2_ and SAD, and its associated threshold (8.0 μg/m^3^), identified in our study offers a meaningful point of comparison with existing literature. While Lee et al., (35) reported a linear association between SO_2_ and SAD in the general Korean population our findings in a high-risk cohort revealed a nonlinear pattern. This discrepancy likely stems from population differences. Individuals at heightened risk for COPD may be more susceptible. They may exhibit a steeper increase in risk (“inflection point”) at lower exposure levels. Additionally, Lee et al., (35) employed a joint-effects model for multiple pollutants, whereas our study examined the independent effect of SO_2_. Nonetheless, both approaches converge in affirming the significant role of SO_2_ in SAD.

A systematic review of 15 studies reported a positive pooled effect for the SO_2_-COPD relationship (*RR* = 1.26), but noted substantial heterogeneity and high risk of bias in most included studies (47). The review highlighted that the prevailing reliance on linear models may obscure true effect thresholds. Our study directly addresses this limitation. For the first time, we reveal a nonlinear exposure-response relationship between SO_2_ and COPD in a high-risk population. We also identify thresholds consistent with those for SAD. This finding offers a novel explanation for the observed heterogeneity: geographic variations in SO_2_ exposure distributions relative to this threshold could lead to systematic differences in effect estimates.

### 4.4 Comparison with previous PM_2.5_ studies

Our nonlinear models quantitatively identified effect thresholds for PM_2.5_ impact on COPD and SAD as 21.33 μg/m^3^, respectively. These values align closely with findings from other Chinese studies. For instance, Li et al. in Tianshui, Gansu, found that COPD hospitalization risk increased significantly when cumulative PM_2.5_ exposure exceeded 25 μg/m^3^ (48), a threshold highly consistent with ours. Taken together, our findings and previous evidence converge to suggest that the 20–25 μg/m^3^ range may represent a critical window for accelerated risk. Conversely, a cross-sectional study in Guangdong used a 35 μg/m^3^ cut-off and found elevated COPD risk (*OR* = 2.416) only above this level (49). The lower threshold identified in our study may reflect the heightened susceptibility of our high-risk cohort.

Evidence for PM_2.5_ thresholds related to SAD is further supported by a nationwide study across 122 Chinese cities (50). Using alveolar nitric oxide (CaNO) as a marker of small airway inflammation (51), they found that the exposure-response slope for PM_2.5_ became statistically significant above 20 μg/m^3^ (50). This finding is highly concordant with our 21.33 μg/m^3^ threshold. Together, these studies implicate the 20–22 μg/m^3^ range as a critical interval for escalating small airway health risks. Mechanistic support comes from an *in vitro* study by Tripathi et al. (40) which demonstrated that low-dose PM_2.5_ ( ≤ 1 μg/m^3^) can upregulate airway disease susceptibility genes such as ADAM33 and ORMDL3. The nonlinear threshold identified in our susceptible population, contrasted with the linear relationship observed in the general population by Lee et al. (35), further suggests that high-risk groups possess greater sensitivity to PM_2.5_.

### 4.5 Comparison with previous SO_2_ studies

For SO_2_, we identified effect thresholds for COPD and SAD at 8.67 μg/m^3^ and 8.67 μg/m^3^, respectively. These values are remarkably low, consistent with inferences from a WHO technical report (52). The report synthesized data from studies in Hong Kong, London, and 12 Canadian cities. It noted that no clear threshold was discernible within the 5–40 μg/m^3^ range, and significant mortality associations persisting even at mean concentrations as low as 5–6.7 μg/m^3^. This implies that if a threshold exists, it must be very low. Our identified threshold of 8.67 μg/m^3^ falls squarely within this very low concentration range, closely matching the WHO's inference. In contrast, a study on AECOPD in Qingyang, Gansu, found that SO_2_ levels exceeding 58 μg/m^3^ significantly increased acute exacerbation risk (9). This substantially higher threshold is likely attributable to differences in outcome measures (prevalence vs. acute events) and population characteristics.

### 4.6 Clinical implications

It is noteworthy that exposure-response relationships can differ across health outcomes. Global Burden of Disease data suggest an approximately linear relationship between PM_2.5_ and COPD mortality, with no discernible safe threshold (a 4.23% mortality increase per IQR increment) (53). The UK Biobank study reported a 1.24% increase in COPD incidence per 10 μg/m^3^ rise in PM_2.5_ (54). Similarly, a Wuhan time-series study found a 1.911% increase in COPD mortality per 10 μg/m^3^ increase in SO_2_ (55), while an Iranian study estimated that 1.2–2.3% of COPD hospitalizations were attributable to SO_2_ (7). These findings complement our threshold estimates, highlighting that different endpoints may yield distinct exposure-response patterns.

The clinical relevance of our thresholds was supported by comparing clinical characteristics between high and low exposure groups. High exposure individuals not only had higher disease prevalence but also exhibited significantly worse symptoms and lung function. Specifically, the high PM_2.5_ group had a higher cough incidence, while the high SO_2_ group reported more dyspnea. These observations are consistent with prior research. A Boston longitudinal study found that each IQR increase in community PM_2.5_ was linked to an 18% higher risk of symptom worsening (*OR* = 1.18, 95% CI*:* 1.02–1.37) (56). The ACURE study reported a 5.4% increase in AECOPD risk per IQR increase in short-term PM_2.5_ (*OR* = 1.054, *95% CI:* 1.012–1.097) (14). A meta-analysis of 37 studies found that each 10 μg/m^3^ increase in PM_2.5_ was associated with a 2.5% increase in COPD-related emergency visits and hospitalizations (95% CI: 1.6%−3.4%) (57). Collectively, this evidence substantiates the persistent threat of particulate pollution, aligning with the symptom burden observed in our high exposure groups.

Regarding lung function, a Wuhan longitudinal study demonstrated that sustained high PM_2.5_ exposure was associated with an additional 2.99% decline in FEV_1_/FVC (95% CI: 0.91–5.08) and an additional 380.15 mL/s decline in PEF (95% CI*:* 32.82–727.48) compared to sustained low exposure (58). These longitudinal data support our cross-sectional results of poorer lung function in the high exposure group, together confirming the progressive nature of lung function impairment due to chronic PM_2.5_ exposure.

SO_2_ exposure was also linked to symptom exacerbation. In our study, dyspnea was significantly more common in the high SO_2_ group, particularly among diagnosed COPD patients (90.0% vs. 83.4%, *P* = 0.016). The meta-analysis by DeVries et al., (57) confirmed a 2.1% increase in COPD-related acute events per 10 μg/m^3^ increase in SO_2_ (95% CI: 0.7%−3.5%). The mechanism is well-established. SO_2_ dissolves in airway lining fluid to form sulfuric and sulfurous acids, lowering pH, directly damaging epithelial cells, activating sensory nerves, and inducing inflammation and oxidative stress (59). Coupled with the ACURE finding that symptomatic patients are more sensitive to pollution, our results underscore that exceeding the SO_2_ threshold significantly increases symptom burden in high-risk populations and COPD patients.

The slight rebound at the highest exposure levels has been observed in other RCS-based studies, where similar patterns were attributed to reduced data density in the upper tail and estimation instability (60). Consistent with these reports, we interpret this rebound as a statistical artifact driven by limited sample size in the high-exposure region and the spline's tail-linear constraint, rather than a genuine biological phenomenon (Supplementary Figure S4, Supplementary Table S3, and Supplementary Figure S5).

### 4.7 Discussion of air pollution and other environmental exposures

COPD arises from complex interactions between genetic susceptibility and long-term environmental exposures, with risk profiles varying markedly by region and population (32). Inhalational exposures are central to its pathogenesis, primarily encompassing tobacco smoke, ambient PM_2.5_, and indoor biomass fuel combustion (11). Our random forest analysis ranked PM_2.5_ and SO_2_ as top contributors to both COPD and SAD, likely reflecting the widespread and persistent nature of ambient air pollution exposure. Smoking is an indisputable risk factor (19, 32). A global ecological study estimated that each IQR increase in tobacco consumption was associated with a 0.30% increase in COPD prevalence and a 0.29% increase in mortality (61, 62). However, the American Thoracic Society notes that the population attributable fraction for smoking varies widely (9.7%−97.9% across studies) and is below 80% in most. This indicates that a substantial portion of the disease burden remains unexplained by smoking alone (33).

Indoor biomass fuel combustion is a critical risk factor in developing countries, where approximately 36% of the global population (2.8 billion) relies on solid fuels for cooking and heating, a figure reaching 61% in rural areas (63). A meta-analysis has affirmed the high epidemiological credibility of the link between biomass smoke and COPD (32). This smoke contains numerous toxic and irritant compounds that can induce airway inflammation, exacerbate symptoms, and accelerate lung function decline (19).

Ambient air pollution, as a ubiquitous exposure, has garnered increasing attention. A systematic review explicitly listed ambient air pollution among 45 non-genetic risk factors for COPD (32). GBD data indicate that air pollution accounts for 8% of all DALYs, a proportion exceeding that attributable to smoking and biomass fuel (10). Despite this, its role in individual risk stratification has often been underestimated. This is likely because its effects are universal, lagged, and cumulative, making direct comparison with active exposures like smoking challenging in traditional epidemiological analyses.

While smoking remains a major risk factor, its population attributable fraction may be lower than previously estimated in contemporary China (19). Concurrently, rapid industrialization and urbanization have rendered outdoor air pollution a pervasive and persistent exposure, elevating its population attributable risk. Unlike smoking (which is modifiable at the individual level) or biomass smoke (which can be reduced through improved stoves), outdoor air pollution is a universal, around-the-clock exposure that is difficult to avoid proactively (33). For never-smokers, it may represent the most important modifiable risk factor; for smokers, it acts synergistically as an “amplifier” of tobacco-induced harm (64). Mechanistically, PM_2.5_ can induce heterogeneous inflammatory responses, a pattern significantly modified by smoking status, with more pronounced systemic effects observed in smokers (65).

In low- and middle-income countries, indoor air pollution may carry a higher population attributable fraction. A multicenter study estimated the population attributable fraction for indoor air pollution at 13.5% (95% CI: 6.4–20.6%), exceeding that for smoking (12.4%) (66). Furthermore, an analysis of GBD 2019 data revealed dynamic shifts in attributable burdens. From 1990 to 2019, the burdens from smoking and biomass fuel declined, while that from ambient particulate matter increased. This increase was particularly notable in low and low-middle SDI regions, where exposure levels rose most sharply (67). This trend, echoed in our findings, underscores the escalating contribution of ambient air pollution to COPD in rapidly developing regions, necessitating its integration into core prevention strategies.

### 4.8 Policy implications

China's clean air policies, such as the “Air Pollution Prevention and Control Action Plan,” have yielded substantial benefits (68). Studies have linked improved air quality to slower lung function decline and reduced COPD risk. A longitudinal study by Bo et al. found that each 5 μg/m^3^/year decrease in PM_2.5_ was associated with a slowed annual decline in FEV_1_ by 19.93 mL (95% CI: 17.42–22.43), FVC by 12.76 mL (95% CI: 9.84–15.66), and MEF_25 − 75_ by 70.22 mL/s (95% CI: 64.69–76.16), along with a 12% reduction in COPD incidence (95% CI: 7%−17%) (69). Therefore, the thresholds identified in our study can inform evidence-based targets for further optimizing clean air policies, ultimately enhancing population respiratory health.

### 4.9 Strengths and limitations

The key innovations of this study are threefold. First, methodologically, we implemented a three-step analytical framework: nonlinear threshold identification, clinical feature evaluation, and multifactor importance ranking. This framework transforms statistical thresholds into clinically meaningful stratification tools. Second, from an evidence perspective, we provide localized exposure-response data for high-risk populations in a plateau region of Southwest China, including novel threshold estimates for SO_2_. Third, practically, our findings offer a scientific basis for developing concentration-based environmental health warnings and targeted protection measures for vulnerable populations in Yunnan Province and analogous regions.

This study has several limitations. First, its cross-sectional design precludes causal inference. Additionally, both threshold identification and clinical characterization were performed within the same dataset and should be interpreted as within-sample comparisons rather than external validation. Accordingly, all relevant stratified findings are merely exploratory and require external verification in independent cohorts before clinical or policy application. Second, individual exposure was assigned using city-level annual average concentrations of PM_2.5_ and SO_2_, which may introduce exposure misclassification due to unmeasured within-city variability. This non-differential misclassification would bias effect estimates toward the null, potentially underestimating the true associations. To address this limitation, we adjusted for major individual-level confounders in all models, including age, sex, smoking status, and household biomass fuel exposure. However, residual confounding from unmeasured city-level factors—such as urban development, industrial structure, and local economic conditions—cannot be entirely ruled out and may have influenced the observed associations. Third, despite adjusting for major confounders, residual confounding from factors like subtle variations in socioeconomic status or occupational exposures cannot be entirely ruled out. Fourth, the small sample size in the high-exposure tail may have contributed to estimation instability at the upper end of the dose–response curves. This pattern should therefore be interpreted cautiously and warrants confirmation in larger studies with more participants in the high-exposure range. Additionally, a key limitation is that both threshold identification and stratified clinical evaluation relied on the same dataset, which may induce overfitting bias.

These limitations point to directions for future research. Longitudinal cohort studies are needed to establish the causality of the identified thresholds. More granular individual-level data collection and methods like Mendelian randomization could further mitigate confounding. Finally, integrating exposomic, metabolomic, and epigenomic approaches may elucidate the specific biological mechanisms linking air pollution to respiratory diseases at an individual level.

## 5 Conclusions

This study demonstrated that individuals with COPD and SAD had significantly higher exposure to PM_2.5_ and SO_2_. Among the high-risk population for COPD, nonlinear exposure-response relationships were identified between the exposure to these two pollutants and the prevalence of COPD and SAD, with effect thresholds of 21.33 μg/m^3^ for PM_2.5_ and 8.67 μg/m^3^ for SO_2_, respectively. Among high-risk individuals and patients with COPD, exposure above these thresholds was associated with more severe respiratory symptoms and poorer lung function.

## Data Availability

The original contributions presented in the study are included in the article/Supplementary material, further inquiries can be directed to the corresponding authors.
