# Supplementary material for: Exposure-response relationship between ambient PM2.5 and SO2 with COPD and SAD prevalence among individuals at high risk for COPD in Yunnan Province, China: a threshold analysis
Source: Front Public Health. 2026 Sep 1;14:1874156. doi: 10.3389/fpubh.2026.1874156 (PMC13574943; doi:10.3389/fpubh.2026.1874156)
Supplement: Supplementary file 1 [file Supplementary_File_1.docx]

Supplementary Figure S1. Sensitivity analysis using 3-knot restricted cubic spline models for COPD and SAD prevalence in relation to PM₂.₅ and SO₂ exposure(3 knots).

Knots were placed at the 10th, 50th, and 90th percentiles. The solid lines represent the adjusted odds ratios, with shaded areas indicating the 95% confidence intervals. Models were adjusted for age, sex, residential altitude, smoking status, and household biomass fuel exposure. Blue triangular markers (▲) along the x-axis indicate knot positions, with corresponding percentile labels (10th, 50th, 90th) shown below. Vertical dashed lines mark the identified threshold concentrations: (A) PM₂.₅ threshold at 21.33 µg/m³ for COPD; (B) SO₂ threshold at 8.67 µg/m³ for COPD; (C) PM₂.₅ threshold at 21.33 µg/m³ for SAD; (D) SO₂ threshold at 8.67 µg/m³ for SAD. The analysis included 2252 high-risk individuals.

Supplementary Figure S2. Sensitivity analysis using 4-knot restricted cubic spline models for COPD and SAD prevalence in relation to PM₂.₅ and SO₂ exposure(4 knots).

Knots were placed at the 5th, 35th, 65th, and 95th percentiles. The solid lines represent the adjusted odds ratios, with shaded areas indicating the 95% confidence intervals. Models were adjusted for age, sex, residential altitude, smoking status, and household biomass fuel exposure. Blue triangular markers (▲) along the x-axis indicate knot positions, with corresponding percentile labels (5th, 35th, 65th, 95th) shown below. Vertical dashed lines mark the identified threshold concentrations: (A) PM₂.₅ threshold at 21.33 µg/m³ for COPD; (B) SO₂ threshold at 8.67 µg/m³ for COPD; (C) PM₂.₅ threshold at 21.33 µg/m³ for SAD; (D) SO₂ threshold at 8.67 µg/m³ for The analysis included 2252 high-risk individuals.

Supplementary Figure S3. Sensitivity analysis using 6-knot restricted cubic spline models for COPD and SAD prevalence in relation to PM₂.₅ and SO₂ exposure(6 knots).

Knots were placed at the 5th, 23rd, 41st, 59th, 77th, and 95th percentiles. The solid lines represent the adjusted odds ratios, with shaded areas indicating the 95% confidence intervals. Models were adjusted for age, sex, residential altitude, smoking status, and household biomass fuel exposure. Blue triangular markers (▲) along the x-axis indicate knot positions, with corresponding percentile labels (5th, 23rd, 41st, 59th, 77th, 95th) shown below. Vertical dashed lines mark the identified threshold concentrations: (A) PM₂.₅ threshold at 21.33 µg/m³ for COPD; (B) SO₂ threshold at 8.67 µg/m³ for COPD; (C) PM₂.₅ threshold at 21.33 µg/m³ for SAD; (D) SO₂ threshold at 8.67 µg/m³ for SAD. The analysis included 2252 high-risk individuals.

Supplementary Figure S4. Exposure distributions of PM₂.₅ and SO₂ among the high-risk population (n = 2,252).

(A) Histogram and density plot of PM₂.₅ exposure with rug marks; (B) Histogram and density plot of SO₂ exposure with rug marks. The solid line represents the kernel density curve. Summary statistics (sample size, 95th percentile, and 97.5th percentile) are displayed within each panel.

Supplementary Figure S5. Restricted cubic spline models for PM₂.₅ and SO₂ exposure with COPD and SAD prevalence among the high-risk population, truncated at the 90th percentile (PM₂.₅: 26.33 μg/m³; SO₂: 10.67 μg/m³).

The solid lines represent the adjusted odds ratios, with shaded areas indicating the 95% confidence intervals. Models were adjusted for age, sex, residential altitude, smoking status, and household biomass fuel exposure. Vertical dashed lines mark the identified threshold concentrations at 21.33 μg/m³ for PM₂.₅ and 8.67 μg/m³ for SO₂. For PM₂.₅ models, truncation at the 90th percentile retained 2,044 participants; for SO₂ models, truncation at the 90th percentile retained 2,176 participants.

Supplementary Figure S6. Bootstrap distributions of threshold estimates (500 replicates).

Density plots showing the distribution of threshold estimates from 500 bootstrap resamples for each of the four models. Red solid line: original threshold (full dataset). Blue dashed line: mean bootstrap threshold. Blue dotted lines: 95% CI bounds. The narrow distributions confirm the stability of the threshold estimates across repeated resampling.
