# Supplementary material for: Exposure-response relationship between ambient PM2.5 and SO2 with COPD and SAD prevalence among individuals at high risk for COPD in Yunnan Province, China: a threshold analysis
Source: Front Public Health. 2026 Sep 1;14:1874156. doi: 10.3389/fpubh.2026.1874156 (PMC13574943; doi:10.3389/fpubh.2026.1874156)
Supplement: Supplementary file 2 [file Supplementary_File_2.docx]

**Supplementary Table S1. Sensitivity analysis of threshold estimates across alternative knot specifications(n=2252).**

| **Specification** | PM₂.₅ → COPD | SO₂ → COPD | PM₂.₅ → SAD | SO₂ → SAD |
| --- | --- | --- | --- | --- |
| 3 knots | 21.33 | 8.67 | 21.33 | 8.67 |
| 4 knots | 21.33 | 8.67 | 21.33 | 8.67 |
| 5 knots (default) | 21.33 | 8.67 | 21.33 | 8.67 |
| 6 knots | 21.33 | 8.67 | 21.33 | 8.67 |

All values are expressed as μg/m³. Thresholds were identified using the OR = 1 crossing point method with full covariate adjustment (age, sex, residential altitude, smoking status, and household biomass fuel exposure).

## Supplementary Table S2. Knot locations for alternative knot specifications(n=2252).

| **Specification** | **Knot Percentiles** | **PM₂.₅ Knot Locations (μg/m³)** | **SO₂ Knot Locations (μg/m³)** |
| --- | --- | --- | --- |
| 3 knots | 10th, 50th, 90th | 16.67, 22.33, 26.33 | 6.00, 9.67, 10.67 |
| 4 knots | 5th, 35th, 65th, 95th | 13.67, 21.33, 24.67, 27.33 | 5.67, 8.33, 10.00, 10.67 |
| 5 knots (default) | 5th, 27.5th, 50th, 72.5th, 95th | 13.67, 21.33, 22.33, 24.67, 27.33 | 5.67, 7.33, 9.67, 10.00, 10.67 |
| 6 knots | 5th, 23rd, 41st, 59th, 77th, 95th | 13.67, 21.00, 21.33, 24.00, 24.67, 27.33 | 5.67, 7.33, 8.67, 9.67, 10.00, 10.67 |

## Supplementary Table S3. Prevalence of COPD and SAD across PM₂.₅ exposure deciles among the high-risk population(n=2252).

| **Decile** | **PM_2.5_ Range** | **COPD prevalence** | **SAD prevalence** |
| --- | --- | --- | --- |
| 1 | 13.67–16.67 | 14.4 | 39 |
| 2 | 16.67–20.67 | 11.9 | 36.3 |
| 3 | 20.67–21.33 | 25.2 | 54.2 |
| 4 | 21.33–21.33 | 25.5 | 53.5 |
| 5 | 21.33–22.33 | 24.3 | 52.2 |
| 6 | 22.33–24.67 | 39.6 | 60.8 |
| 7 | 24.67–24.67 | 41.3 | 62.1 |
| 8 | 24.67–24.67 | 41.3 | 62.1 |
| 9 | 24.67–26.33 | 40.1 | 63.9 |
| 10 | 26.33–27.33 | 21.2 | 53.7 |

## Supplementary Table S4. Prevalence of COPD and SAD across SO₂ exposure deciles among the high-risk population(n=2252).

| **Decile** | **SO_2_ Range** | **COPD prevalence** | **SAD prevalence** |
| --- | --- | --- | --- |
| 1 | 5–6 | 11.9 | 37.6 |
| 2 | 6–6.67 | 20.5 | 46.2 |
| 3 | 6.67–8.33 | 25 | 46.8 |
| 4 | 8.33–8.67 | 30.7 | 61.7 |
| 5 | 8.67–9.67 | 32.6 | 64.5 |
| 6 | 9.67–9.67 | 31.6 | 57.1 |
| 7 | 9.67–10 | 28.8 | 53.2 |
| 8 | 10–10 | 26.5 | 50 |
| 9 | 10–10.67 | 29.6 | 55.4 |
| 10 | 10.67–12.67 | 35.1 | 63.8 |

**Supplementary Table S5. Bootstrap validation of threshold estimates (500 replicates,n=2252)**

| Model | Original Threshold | Mean Bootstrap±SD | 95% CI | Valid Iterations |
| --- | --- | --- | --- | --- |
| PM_2.5_→COPD | 21.33 | 22.26±0.51 | 21.33-24 | 500 |
| SO_2_→COPD | 8.67 | 9.63±0.13 | 9.33-9.67 | 500 |
| PM_2.5_→SAD | 21.33 | 22.29±0.59 | 21.33-24 | 500 |
| SO_2_→SAD | 8.67 | 9.63±0.19 | 9.33-9.67 | 500 |

**Supplementary Table S6. Sensitivity analyses for age restriction and smoking adjustment.**

| Model | Primary Analysis(n=2252) | Age ≥40 Years Subgroup(n=2039) | Without Smoking Adjustment(n=2252) |
| --- | --- | --- | --- |
| PM2.5→COPD | 21.33 | 22.33 | 21.33 |
| SO2→COPD | 8.67 | 9.67 | 8.67 |
| PM2.5→SAD | 21.33 | 22.33 | 21.33 |
| SO2→SAD | 8.67 | 9.67 | 8.67 |

All values are presented as threshold estimates (μg/m³). Primary analysis: full dataset with all covariates adjusted (age, sex, residential altitude, smoking status, and household biomass fuel exposure). Age ≥40 years: restricted to participants aged ≥40 years; age was not included as a covariate because the subgroup was already age-restricted; models were adjusted for sex, residential altitude, smoking status, and household biomass fuel exposure.
